# Optimal Replacement of Soybean Meal with Fermented Palm Kernel Meal as Protein Source in a Fish Meal-Soybean Meal-Based Diet of Sex Reversed Red Tilapia (*Oreochromis niloticus* × *O. mossambicus*)

**DOI:** 10.3390/ani11082287

**Published:** 2021-08-03

**Authors:** Wattana Wattanakul, Karun Thongprajukaew, Waraporn Hahor, Naraid Suanyuk

**Affiliations:** 1 Department of Aquaculture and Fisheries Products, Faculty of Science and Fisheries Technology, Rajamangala University of Technology Srivijaya, Trang 92150, Thailand; 2 Division of Health and Applied Sciences, Faculty of Science, Prince of Songkla University, Songkhla 90112, Thailand; karun.t@psu.ac.th (K.T.); tarn.something@gmail.com (W.H.); 3 Kidchakan Supamattaya Aquatic Animal Health Research Center, Aquatic Science and Innovative Management Division, Faculty of Natural Resources, Prince of Songkla University, Songkhla 90112, Thailand; naraid.s@psu.ac.th

**Keywords:** carcass, digestive enzyme, feed utilization, flesh quality, hematological parameter, liver

## Abstract

**Simple Summary:**

Replacement effects of soybean meal (SBM) with fermented palm kernel meal (FPKM) as a protein source was investigated in sex-reversed red tilapia. The two-month-old fish were fed fish meal-SBM-based diets with replacement by FPKM at 25% (25FPKM), 50% (50FPKM), 75% (75FPKM) and 100% (100FPKM) for 12 weeks, while an FPKM-free diet (0FPKM) was used as a control. Based on growth performance, feed utilization, digestive enzyme activities, flesh quality, carcass composition, hematological parameters and liver histoarchitecture, the 50% replacement level of SBM by FPKM support this alternative. Findings from the current study support the use of FPKM in aquafeed production, providing a low-cost diet for tilapia farming.

**Abstract:**

The solid-state fermentation by effective microorganisms (containing photosynthetic bacteria, lactic acid bacteria, nitrogen-fixing bacteria, yeast and *Bacillus* sp.) improved the nutritive values of palm kernel meal (PKM). Increased crude protein (20.79%), nitrogen-free extract (40.07%) and gross energy (19.58%) were observed in fermented PKM (FPKM) relative to raw PKM while crude lipid (15.65%), crude fiber (36.45%) and ash (29.54%) were decreased. Replacement of soybean meal (SBM) with FPKM as a protein source was investigated for its effects in sex-reversed red tilapia (*Oreochromis niloticus* × *O. mossambicus*). The two-month-old fish (14.85 ± 0.28 g initial weight) were fed fish meal-SBM-based diets with replacement by FPKM at 25% (25FPKM), 50% (50FPKM), 75% (75FPKM) and 100% (100FPKM), while an FPKM-free diet (0FPKM) was used as a control. The five treatments, comprising triplicate cement ponds and forty fish each, were conducted in a recirculating system over 12 weeks. At the end of the feeding trial, fish fed the 50FPKM diet were superior in growth performance, while the feed utilization parameters were similar across all five treatments. Physiological adaptation of the protein-digesting (pepsin and trypsin) and lipid-digesting (lipase) enzymes was detected at all protein replacement levels (except for 25FPKM), as well as of the enzyme for cellulose digestion (cellulase), but not of the carbohydrate-digesting enzymes (amylase). Protein synthesis capacity in flesh was improved in fish fed the 50FPKM diet, while the quality of the main flesh proteins, actin and myosin, showed no significant differences across the five treatments. No differences in carcass composition and no negative effects on hematological parameters or liver histoarchitecture at the 50% replacement level of SBM by FPKM also support this alternative. Findings from the current study indicate the low-cost FPKM-containing diet for tilapia in comparison with control diet.

## 1. Introduction

Nile tilapia (*Oreochromis niloticus*) is an economically important fish species that is widely cultured around the world [1]. These fish are a good alternative protein source for human consumption, especially in tropical and subtropical zones [2]. Red tilapia (*O. niloticus* × *O. mossambicus*) is popular due to its attractive color and good taste, increasing its marketability [3]. Similar to other economic fish species, the cost of main protein ingredients (mainly fish meal) for formulating the pellet diets is continuously increasing [4]. Therefore, the protein sources from plant by-products have been used to replace fish meal in fish diets [5,6], as have low-cost animal by-products [7,8]. 

Soybean meal (SBM) is the main plant protein in practical fish feed production. This feedstuff is moderately rich in protein, produced in great quantities, reasonably priced, and has relatively well-balanced amino acid profile among the plant by-product meals [9,10]. The presence of anti-nutritional compounds (including goitrin, phytohemagglutinins, lectins, non-starch polysaccharides, phytate, phytoestrogens, protein antigens, saponins and trypsin inhibitor) are disadvantages of this plant by-product in aquafeed production [11,12]. Therefore, the replacement of SBM by low-cost plant protein sources is worth considering in the aquaculture sector. 

Palm kernel meal (PKM) is mainly produced in South-East Asian and African countries. This by-product is obtained from palm kernel oil extraction processes and is generally used in feeds for terrestrial animals [13,14] and as a component in fish feed formulations [15,16,17,18]. Poor usability in aquafeed is due to the large amount of cell wall constituents, low protein content and amino acid deficiencies [15,16]. Biological pretreatment of the PKM for improving its nutritive value appears to have potential, via enzyme supplementation and fermentation by cellulolytic or cocktail enzymes [14,15,16].

Effective microorganisms (EMs) are various blends of common predominantly anaerobic microorganisms, probably including lactic acid bacteria (LAB), photosynthetic bacteria, yeast and naturally beneficial microorganisms. Worldwide, EMs support sustainable practices in farming, composting and mitigation of environmental pollution. In animal nutrition research, EMs have been applied to benefit economic terrestrial and aquatic animals [19]. Various enzymes produced by the EMs might improve the nutritive value of PKM. Therefore, replacement effects of SBM protein by fermented PKM (FPKM) were investigated in sex-reversed red tilapia in the current study. The commercially available EM (EM-Plus, Microbe for Life, Bangkok, Thailand) that contains photosynthetic bacteria, LAB, nitrogen-fixing bacteria, yeast and *Bacillus* sp. was used in the current study since it is widely used by Thai farmers. The suitable replacement levels were assessed from growth performance, feed utilization, digestive enzyme specific activities, flesh quality, carcass composition, hematological parameters and liver histoarchitecture. 

## 2. Materials and Methods

### 2.1. Preparation of FPKM

The PKM was obtained from Phatthalung Livestock CO., LTD, Phatthalung, Thailand. This by-product was fermented with activated effective microorganisms (EM-Plus, Microbe for Life, Bangkok, Thailand) that contain photosynthetic bacteria, LAB, nitrogen-fixing bacteria, yeast and *Bacillus* sp. The solid-state fermentation was performed by mixing PKM with liquid EM (5% *v*/*w*), molasses (5% *v*/*w*) and then adding water (5% *v*/*w*). These mixtures were packed in polyethylene bags, sealed and incubated for 30 days in dark at ambient temperatures (30–31 °C). 

### 2.2. Preparation of the Experimental Diets 

The formulations and ingredients of the experimental diets are shown in Table 1. All the experimental diets were formulated to contain approximately 30% crude protein and 10% crude lipid on averages. The protein from SBM was replaced by FPKM at 25% (25FPKM), 50% (50FPKM), 75% (75FPKM) and 100% (100FPKM), while an FPKM-free diet (0FPKM) was used as the control. Fish meal and SBM were used as main protein sources, while carbohydrate sources included corn meal, broken rice and rice bran. All dry ingredients were finely ground to 80 mesh particle size, weighed accurately and then mixed for 10 min in a mixer. Pre-weighed lipid sources (fish oil and soybean oil) were slowly blended, followed by vitamin-mineral premixes and 30% by weight of distilled water. The glutinous mixture was passed through a meat mincer (4–6 mm), dried at 60 °C for 24 h, sifted to remove any fine particles and then kept in polyethylene bags at 4 °C until feeding. 

### 2.3. Proximate Compositions of Ingredients and Experimental Diets 

Proximate chemical compositions of ingredients (PKM and FPKM) and experimental diets were analyzed for moisture, crude protein, crude lipid, crude fiber and ash, according to standard methods of AOAC [20]. Nitrogen-free extract (NFE) and gross energy (GE, kJ g^−1^) were calculated as [100 − (moisture + crude protein + crude lipid + crude fiber + ash)] and as [(crude protein × 23.6) + (NFE × 17.2) + (crude lipid × 39.5)], respectively. All the chemical analyses were performed in duplicates and are expressed as % dry matter.

### 2.4. Fish Feeding Trial 

Transportation, husbandry, feeding trial and sampling of animals in the current study conformed to the “Ethical Principles and Guidelines for the Use of Animals for Scientific Purposes”, National Research Council, Thailand (Application No. UI-02771-2559 and U1-06514-2560). Two-month-old sex-reversed red tilapia were purchased from Phatthalung Inland Fisheries Research and Development Center, Phatthalung, Thailand. The fish were acclimatized for 10 days in a 4000 L cement pond (1 m × 4 m × 1 m) and fed with 0FPKM diet twice daily (08.00 and 16.00 h) to satiation. Subsequently, forty fish each (14.85 ± 0.28 g initial weight) were randomly distributed into cement ponds (1 m × 2 m × 0.6 m) with 40 cm water depth. There were fifteen experimental units in total—five treatments with three replicates each. The fish were fed with 10% of body weight (BW) per day and the feed amount was adjusted weekly according to the actual feeding performance. Experimentation was conducted under a 12 h light/12 h dark natural light cycle. Recirculating aquaculture system was used, controlling the water quality to pH 7.91 ± 0.06, 28.73 ± 0.22 °C temperature, 6.11 ± 0.11 mg L^−1^ dissolved oxygen, 97.80 ± 1.47 mg L^−1^ alkalinity, 0.54 ± 0.04 mg L^−1^ ammonia and 0.61 ± 0.03 mg L^−1^ nitrite. Uneaten feed was collected 1 h after feeding, dried at 60 °C until constant weight and used to calculate the feeding rate (FR), the feed conversion ratio (FCR) and the protein efficiency ratio (PER). At the end of 12 weeks of trial, all the fish were starved for 24 h, anesthetized by quinaldine and then body weight and length were measured for every fish. Nine fish from each dietary treatment were harvested, minced and then stored at –20 °C until use for whole carcass proximate composition analysis. Nine samples from the remaining fish were used for collecting blood, stomach, intestine, liver and white muscle. Blood samples were collected from the caudal vessel after anesthetization. The samples were kept at 4 °C and all hematological parameters were determined within 12 h after collection. The fish were dissected by sterilized scalpels on ice and then stomach and intestinal samples were carefully removed and stored at –20 °C until use for digestive enzyme assay. Liver samples were immediately removed and then fixed in 10% neutral-buffered formalin. The skin and scales were removed before collecting white muscle (epaxial muscle below the dorsal fin) for flesh quality assessment. Growth performance and feed utilization parameters were calculated as follows:

Survival (%) = 100 × [Final fish number/initial fish number]

Fulton’s condition factor (*K*) = 100 × [Live body weight (g)/total body length (cm)^3^]

Daily growth coefficient (DGC, % BW day^−1^) = 100 × [(W_t_)^1/3^ − (W_0_)^1/3^/(t − t_0_)]

where W_t_ = mean weight (g) at day t, W_0_ = mean weight (g) at day t_0._

Feeding rate (FR, % BW day^−1^) = C/[(W_0_ + W_t_)/2]/t × 100

where C = daily feed consumption (g), W_0_ = initial body weight (g), W_t_ = final body weight (g), t = feeding duration (day).

Feed conversion ratio (FCR, g feed g gain^−1^) = Dry feed consumed (g)/wet weight gain (g)

Protein efficiency ratio (PER, g gain g protein^−1^) = Wet weight gain (g)/protein intake (g)

### 2.5. Digestive Enzyme Studies 

Crude extract from the frozen digestive organs (stomach or intestine) were prepared by homogenizing the target tissues in 0.2 M Na_2_HPO_4_-NaH_2_PO_4_ buffer at pH 8 (1:3 *w/v*) using a micro-homogenizer (THP-220; Omni International, Kennesaw, GA, USA). The homogenate was centrifuged at 15,000× *g* for 30 min at 4 °C, and then the supernatant was collected and kept at −20 °C until use. The protein concentration in crude enzyme extract was assayed using the method of Lowry et al. [21] within the linear range for standard bovine serum albumin (BSA). The concentration of the soluble protein (mg mL^−1^) was used to quantify the enzyme specific activities (U mg protein^−1^). All assays were performed within one month after extraction.

Pepsin (EC 3.4.23.1) activity from stomach extracts was determined based on the method of Worthington [22], with some modifications. First, 500 µL of 2% hemoglobin (dissolved in 0.06 N hydrochloric acid) was mixed with 100 µL of crude enzyme extract. The reaction mixture was incubated at 55 °C for 10 min and then stopped by adding 1 mL of 5% trichloroacetic acid. The mixture was centrifuged at 12,000× *g* at room temperature for 5 min. Supernatant was collected and was measured spectrophotometrically at 280 nm. One unit (U) of pepsin activity was defined as 1.0 increase in absorbance at 280 nm. 

Trypsin (EC 3.4.21.4) activity from the intestinal extracts was assayed according to Rungruangsak-Torrissen et al. [23], using *N*-benzoyl-*L*-Arg-*p*-nitroanilide (BAPNA) as the substrates. The assay was performed by mixing 700 µL of 0.2 M Na_2_CO_3_-NaHCO_3_ buffer (pH 9) containing 1.25 mM BAPNA with 100 µL of a crude enzyme extract. The mixture was incubated at 50 °C for 10 min and then stopped by adding 800 µL of 30% acetic acid. The absorbance was measured spectrophotometrically at 410 nm and compared with linear range response to *p*-nitroanilide. One unit of trypsin activity was defined as the liberation of 1 μmol of *p*-nitroanilide per min.

Amylase activity (EC 3.2.1.1) was determined based on the method of Areekijseree et al. [24], using soluble starch as the substrate. Briefly, 25 µL of 5% soluble starch, 62.5 µL of 0.2 M Na_2_HPO_4_-NaH_2_PO_4_ buffer (pH 7), 37.5 µL of 20 mM sodium chloride and 125 µL of crude enzyme extract were mixed and then incubated at 50 °C for 15 min. Subsequently, 250 µL of 1% dinitrosalicylic acid was added to stop the enzyme reaction. The color was developed after boiling at 100 °C for 5 min, cooling to room temperature, and mixing with 2.5 mL of distilled water. Liberated product was measured spectrophotometrically at 540 nm against linear range of standard maltose. One unit of amylase activity was defined as the liberation of 1 μmol of maltose per min. 

Cellulase activity (EC 3.2.1.4) was assayed according to the method of Mendels and Weber [25] with some modifications. The reaction was initiated by mixing 25 µL of 2% carboxymethylcellulose, 62.5 µL of 0.2 M Na_2_HPO_4_-NaH_2_PO_4_ buffer (pH 7) and 37.5 µL of 20 mM sodium chloride with 125 µL of crude enzyme extract. Enzymatic reaction was incubated at 50 °C for 15 min, and then stopped by adding 250 µL of 1% dinitrosalicylic acid. The color was developed after boiling at 100 °C for 5 min, cooling to room temperature and mixing with 2.5 mL of distilled water. Comparison with the linear range of glucose standards was performed at 540 nm in order to calculate the enzyme activity. One unit of cellulase activity was defined as the liberation of 1 μmol of glucose per min. 

Lipase activity (EC 3.1.1.3) was assayed using *p*-nitrophenyl palmitate as substrate, according to the method of Winkler and Stuckmann [26], with some modifications. The reaction mixture contained 200 µL of 0.01 M *p*-nitrophenyl palmitate, 800 µL of 0.2 M Na_2_HPO_4_-NaH_2_PO_4_ buffer (pH 8) and 20 µL of crude enzyme extract. The incubation was performed at 60 °C for 30 min and then stopped by adding 250 µL of 1 M sodium carbonate. The supernatant collection was performed after centrifugation at 13,000× *g* for 15 min at 4 °C. The liberated product was measured spectrophotometrically at 410 nm and referenced to the linear range of *p*-nitrophenol standard. One unit of lipase activity was defined as the liberation of 1 μmol of *p*-nitrophenol per min.

### 2.6. Flesh Quality 

#### 2.6.1. Protein Synthesis Capacity 

RNA and protein concentrations were determined as described by Rungruangsak-Torrissen et al. [23]. Briefly, fifty milligrams of frozen white muscle were mixed with TRIzol^®^ reagent (Invitrogen, Carlsbad, CA, USA) and sonicated (VCX; Sonic and Materials Inc., Newtown, CT, USA) to obtain a pink transparent solution. The mixture was mixed with chloroform and then centrifuged to obtain upper (RNA) and lower (protein) phases. Isopropanol and ethanol were used to precipitate these phases. The RNA sediments were dissolved in sodium acetate and dried at 55 °C while sodium dodecyl sulfate was applied to the protein sediments. RNA and protein concentrations were measured spectrophotometrically at 260 and 280 nm. The measured absorbances were calculated to the concentrations of RNA and protein from the equations *E*_260_ = 40 µg mL^−1^ and *E*_280_ = 2.1 mg mL^−1^, respectively. 

#### 2.6.2. Enthalpy of Actin and Myosin 

Ten milligrams of defrosted white muscle were heated in a differential scanning calorimeter (DSC7; Perkin Elmer, Waltham, MA, USA) from 20 °C to 100 °C at a rate of 10 °C min^−1^. The enthalpic response (ΔH) was automatically recorded to identify actin and myosin based on onset (T_o)_, peak (T_p_) and conclusion (T_c_) temperatures, as reported by Nonthaput et al. [27]. 

### 2.7. Carcass Composition Analysis 

Whole fish were minced and analyzed for moisture, crude protein, crude lipid and crude ash, according to standard methods of AOAC [20]. 

### 2.8. Hematological Determinations 

Blood suspension was prepared [28], and red (RBC) and white (WBC) blood cells were counted with a hemacytometer (Precicolor; HBG, Giessen-Luetzellinden, Germany) under a compound microscope. Hemoglobin (Hb) and hematocrit (Hct) were determined by measuring the formation of cyanmethemoglobin [29] and by using laboratory-prepared capillary tubes treated with 10% heparin [30], respectively. Differential leucocytes were counted from dried blood smears after fixing with methanol and staining with Jenner–Giemsa. Heparinized blood samples were centrifuged at 2000× *g* for 10 min at 4 °C for preparing fish plasma. The plasma protein was determined according to Lowry et al. [21] using BSA as protein standard. Some blood samples were added into non-EDTA tube (non-ethylenediaminetetraacetate) and allowed to clot at 4 °C. The serum samples were kept after centrifugation at 3000× *g* for 10 min at 4 °C. Blood urea nitrogen (BUN), creatinine, uric acid, alkaline phosphatase (ALP) and aspartate aminotransaminase (AST) were determined from the serum samples using a commercial diagnostic kit (PZ Cormay S.A. Company, Lomianki, Poland). Mean cell volume (MCV), mean cell hemoglobin (MCH) and mean cell hemoglobin concentration (MCHC) were calculated as described by Javed et al. [31]:

MCV (fL) = 10 × [Hct (%)/RBC (×10^6^ cells μL^−1^)]

MCH (pg) = 10 × [Hb (g dL^−1^)/RBC (×10^6^ cells μL^−1^)]

MCHC (g dL^−1^) = 100 × [Hb (g dL^−1^)/Hct (%)]

### 2.9. Liver Histological Examination 

Fish liver was collected and examined for histological study using the standard method of Suvarna et al. [32]. After fixation in 10% neutral-buffered formalin and dehydrated through in an ascending ethanol series (70%, 95% and 100%), the samples were embedded in paraffin and 3–5 µm longitudinal sections were cut using a tissue microtome. Liver histology was examined under a light microscope after staining with hematoxylin and eosin (H & E). 

### 2.10. Statistical Analysis 

Data are expressed as mean ± standard error of mean (SEM). All the data were analyzed using SPSS version 17 software (SPSS Inc., Chicago, IL, USA). Arcsine transformation was applied to transform the variables that are percentages. One-way analysis of variance was used, and the mean comparisons were carried out using Duncan’s multiple range test as a post-hoc test at a significance level of α = 0.05 (*p* ˂ 0.05).

## 3. Results

### 3.1. Chemical Compositions of PKM and FPKM 

Fermentation of PKM with EM improved the amount of crude protein (20.79%), NFE (40.07%) and GE (19.58%) while crude lipid (15.65%), crude fiber (36.45%) and ash (29.54%) were decreased (Table 2). 

### 3.2. Survival, Growth and Feed Consumption 

No significant differences in survival (93% on average) were observed across the five dietary treatments (*p* > 0.05, Table 3). Fish fed 50FPKM only presented significant differences from the other treatments in terms of final body weight (and total length with fish fed 25FPKM), although the DGC was not significantly different. Fish fed 25FPKM diet had significantly highest *K* values relative to the other treatments, except for fish fed 50FPKM diet. Feed utilization parameters (FR, FCR and PER) were also similar across the five alternative treatments.

### 3.3. Digestive Enzyme Specific Activities 

Different trends were observed for the digestive enzymes across the five dietary treatments (Table 4). Fish fed 50FPKM and 75FPKM diets had the highest pepsin specific activities relative among the treatments (*p* < 0.05). Trypsin specific activity was highest in the fish fed with the full replacement of SBM by FPKM, followed by the 50% replacement. There was no effect from replacement of SBM by FPKM on the specific activity of amylase. The fish fed 75FPKM and 100FPKM diets had the highest cellulase and lipase activities, while the remaining treatments were mutually rather similar.

### 3.4. Flesh Quality 

RNA concentrations had the highest values in the fish fed 0FPKM, 25FPKM and 50FPKM diets, while significantly increased protein concentrations were observed in fish fed 50FPKM diet relative to the control treatment (Table 5). The RNA/protein ratio (protein synthesis) was highest in the fish fed 0FPKM and was significantly decreased in the fish fed FPKM-containing diets, except for fish fed the 25FPKM diet. The ∆H of myosin, actin, and their sum were similar across the five dietary treatments, while the ∆H ratio of actin to myosin was significantly decreased in the fish fed the 75FPKM diet from that with the control diet. 

### 3.5. Carcass Composition 

At the end of experiment, the carcass moisture, crude protein, crude lipid and ash did not differ across the five dietary treatments (Table 6). 

### 3.6. Hematological Parameters 

WBC was comparatively high in the fish fed with diets replacing at least 50% of SBM by FPKM, relative to the other treatment groups (Table 7). Significantly decreased MCHC was only observed in the fish fed the 75FPKM diet, relative to the other treatments, except for 50FPKM. Partial or full replacement of SBM by FPKM can significantly increase plasma protein. Except for the above parameters, the other hematological assays were very similar across the five dietary treatments.

### 3.7. Liver Histoarchitecture 

**Figure 1:**
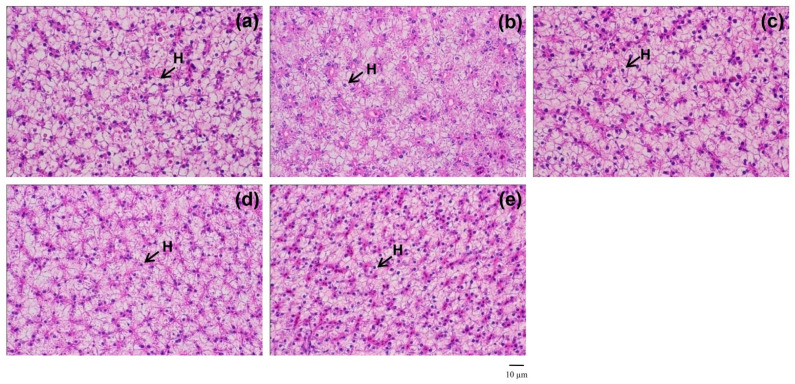
The microanatomy in longitudinal section of liver for sex-reversed red tilapia fed with 0FPKM (**a**), 25FPKM (**b**), 50FPKM (**c**), 75FPKM (**d**) and 100FPKM (**e**) for 12 weeks. Images were taken at 400× magnification and the tissues were stained by hematoxylin and eosin (H&E). H indicates hepatocyte.

Sex-reversed red tilapia fed with an FPKM-free diet (Figure 1a) and with the FPKM-containing diets (Figure 1b–e) exhibited normal-shaped hepatocytes and clearly located cell nuclei. There were no signs of necrosis or inflammation in any fish.

## 4. Discussion

Fermentation of plant by-products with beneficial microorganisms has been adopted to improve the nutritional quality of feedstuffs by the action of enzymes from bacteria, yeasts and molds. Increased protein in FPKM in the current study could be due to the secretion of enzymes or to the release of bound proteins by the breakdown of protein complexes. This presumption is in agreement with increasing protein content in FPKM after conducting spontaneous fermentation for one week (23.42%) relative to raw PKM (20.04%) [33]. It is possible that the replacement of bound proteins from SBM in the current study by amino acids or small proteins from FPKM might improve the diet quality, although the experimental diets were isonitrogenous. In palm kernel cake, a slight increase in protein content (16.43% vs. 16.80%) and significantly improved amino acid profiles have also been observed when fermented with *Paenibacillus polymyxa* ATCC 842: isoleucine (0.50% vs. 0.59%), phenylalanine (0.57% vs. 0.66%), threonine (0.41% vs. 0.51%), histidine (0.23% vs. 0.29%), methionine (0.22% vs. 0.27%), arginine (1.60% vs. 1.76%), glycine (0.60% vs. 0.78%), aspartic acid (1.12% vs. 1.27%), glutamic acid (2.48% vs. 2.80%), proline (0.44% vs. 0.59%) and serine (0.56% vs. 0.69%) [34]. The cell wall constituents and non-starch polysaccharides (NSP) contribute 73% and 75% of raw PKM, respectively [35]. Significantly decreased crude fiber was not surprising since the EM contained cellulolytic microorganisms providing high cellulase activity [36]. Fluctuation in the amount of this indigestible element can increase the digestible carbohydrates, NFE. Fermentation of a medicinal plant with *Lactobacillus plantarum* and *Saccharomyces cerevisiae* can reduce the amount of saturated fatty acids [37]. Decreased lipid content in the current study is possible since the fatty acids in PKM are mainly in saturated forms. Regarding ash, the reduction in FPKM when compared with non-fermented PKM might be caused by the high capacity to utilize the constituent minerals during fermentation [38]. 

Various plant by-products have been used to replace protein from SBM in fish diets. In hybrid tilapia (*O. niloticus* × *O. aureus*), 60% replacement of SBM by cottonseed meal in diets was optimal [5]. Thirty percent replacement of SBM by rubber seed meal was also suitable for the same species [39], similar to the replacement by faba beans at 24% in *O. niloticus* diet [40], while 50% replacement level was suitable by cottonseed meal in diets for channel catfish, *Ictalurus puctatus* [41]. In the current study, sex-reversed red tilapia fed a 50FPKM diet were superior in growth performance, while the feed utilization parameters were similar to the other treatments. However, higher replacement than the optimal level tends to provide adverse effects on growth, feed utilization and health status in several fish species.

Proteolytic activities were investigated through the digestive enzymes, which are mainly present in the stomach (pepsin) and the intestine (trypsin). That the highest overall specific activity was in fish fed the 50FPKM diet, followed by 75FPKM, indicating high capacity to utilize protein from diets. Generally, native PKM contains a large fraction of NSPs and these cell wall constituents inhibit nutrient utilization by encapsulating them in the gastrointestinal tract [35,42]. Significant increase in trypsin specific activity alone, in the fish fed the full replacement diet, might be due to overproduction of enzymes to achieve sufficient protein utilization [43], since approximately 40–50% of the ingested dietary proteins are digested by trypsin [44]. The amylase specific activity was unaffected by the replacement level of SBM by FPKM in the current study, indicating that this omnivorous fish had sufficient access to carbohydrate digestion. Generally, glucose is the primary energy source for a number of tissues [45], so maintenance of its metabolic homeostasis is important. Activity of cellulase increased with the replacement level by FPKM. The up-regulation of this enzyme activity might improve the bioavailability of fiber along the alimentary tract of the reared fish. Lipase activity was generally similar, except in the 75FPKM treatment group. Fluctuation in the fatty acid profiles or their content may alter the lipid utilization. Based on the overall enzyme specific activities, the maintenance of feed utilization parameters (FR, FCR and PER) in the fish fed 75FPKM or 100FPKM diets might consider a protein-sparing effect from cellulose and lipid digestion, as indicated by increased specific activities of fiber- and lipid-digesting enzymes.

White muscle is significantly responsible for metabolism and protein growth [46]. High concentrations of RNA and protein and low protein synthesis capacity (RNA/protein ratio) indicate superior growth performance [18,47] of the fish fed the 50FPKM diet. The quality of flesh actin and myosin was also investigated in the current study. The enthalpy response is associated with the amount of protein left in its native state [48]. Maintaining the four observed enthalpy parameters also supports unchanged quality of the main muscle proteins across the five dietary treatments, except in the fish fed the 75FPKM diet. In addition, no differences were observed in whole carcass composition. This indicates that the fish can defend their composition in the face of feedstuff replacement, which appears to be more important than accelerating growth (DGC) or feed utilization (FR, FCR and PER) [43]. 

Three hematological parameters of the overall sixteen items were affected by the replacement of SBM with FPKM. At least 50% replacement can significantly increase WBC. This increment is associated with lymphopoiesis stimulation, as the leucocytes are the non-specific immune system in fish [49]. The β-glucan from yeast cell wall and *Bacillus* sp. in the EM may act as prebiotics and probiotics, respectively, stimulating the fish immune response. Our results are in agreement with those from replacing SBM with 10–30% rubber seed meal in the diet of rohu fingerlings, *Labeo rohita* [50], or replacement with 15–30% cottonseed meal in the diet of hybrid tilapia [5]. Plasma protein also increased in a replacement level-dependent manner. Fermentation by the EMs might provide easily digested protein to the feed, or a high amount of fish meal can enhance protein digestibility [51], with digestion mainly by trypsin. Replacement of highly digestible fish meal by poorly digestible plant mixtures (corn gluten, wheat gluten, extruded peas, rapeseed meal and sweet white lupin) can consequently decrease the plasma protein level in gilthead sea bream, *Sparus aurata* [52]. The slight changes in the current study are within the standard range of healthy fish, suggesting no negative effects on health status by the preferred treatment. This lack of negative effects is also supported by the unchanged liver histoarchitectures of fish fed the 50FPKM diet (or any of the other treatments tested).

## 5. Conclusions

The nutritive value of palm kernel meal was improved by solid-state fermentation with effective microorganisms. This fermented feedstuff could be used to replace soybean meal at 50%, as indicated by superior growth performance and maintained feed utilization. Some improvements in digestive enzyme activities and flesh protein synthesis capacity were observed, while no negative effects on carcass composition, hematological parameters or liver histoarchitecture also supported this replacement level of soybean meal with fermented palm kernel meal for the reared fish. The low current purchasing price of fermented palm kernel meal (0.32 USD kg^−1^) relative to soybean meal (0.57 USD kg^−1^) supports its use for economic benefits. Findings from the current study support the use of fermented palm kernel meal in aquafeed production, providing a low-cost diet for tilapia farming.

## Figures and Tables

**Table 1:** Formulations and proximate compositions of experimental diets containing varying levels of FPKM.

Item	0FPKM	25FPKM	50FPKM	75FPKM	100FPKM
Ingredient					
Fish meal	26.9	30.8	34.8	38.8	42.7
SBM	25.0	18.8	12.5	6.2	–
FPKM	–	6.2	12.5	18.8	25.0
Corn meal	15.9	14.2	12.5	10.8	9.1
Broken rice	12.7	11.4	10.0	8.6	7.3
Rice bran	8.5	7.6	6.7	5.8	4.9
Fish oil	2	2	2	2	2
Soybean oil	2	2	2	2	2
Alfa starch	4	4	4	4	4
Vitamin-mineral premix ^a^	3	3	3	3	3
Proximate composition (% fed basis)					
Moisture	5.84	5.96	5.81	5.90	5.91
Crude protein	30.63	29.87	30.61	30.22	30.05
Crude lipid	10.13	10.90	10.11	9.54	9.50
Crude ash	13.28	14.50	15.37	17.22	18.78
Crude fiber	7.28	7.47	7.82	8.03	8.98
NFE	32.84	31.30	30.28	29.09	26.78
GE (kJ g^−1^)	16.88	16.74	16.43	15.90	14.45

SBM, soybean meal; FPKM, fermented palm kernel meal; NFE, nitrogen-free extract; GE, gross energy. ^a^ Vitamin-mineral premix, 1 kg contained 1000 U vitamin A, 250 U vitamin D_3_, 5 U vitamin E, 2000 mg vitamin B_1_, 800 mg vitamin B_2_, 2000 mg vitamin B_6_, 1 mg vitamin B_12_, 10,000 mg vitamin C, 300 mg pantothenic acid, 5000 mg nicotinic acid, 200 mg folic acid, 2 mg biotin, 500 mg iron, 7000 mg zinc, 2 mg biotin, 800 mg manganese, 10 mg selenium, 15,000 mg lysine, 3000 mg methionine.

**Table 2:** Proximate chemical compositions (% of dry matter) of PKM and FPKM. The data given are means from duplicate analysis.

Composition	PKM	FPKM
Crude protein (%)	13.13	15.86
Crude lipid (%)	9.14	7.71
Crude fiber (%)	38.74	24.62
Ash (%)	4.03	2.84
NFE (%)	34.96	48.97
GE (kJ g^−1^)	12.72	15.21

PKM, palm kernel meal; FPKM, fermented palm kernel meal; NFE, nitrogen-free extract; GE, gross energy.

**Table 3:** Survival, growth performance and feed utilization of sex-reversed red tilapia fed with experimental diets containing varying levels of FPKM for 12 weeks.

Parameter	0FPKM	25FPKM	50FPKM	75FPKM	100FPKM	*p*-Value
Survival (%)	93.13 ± 1.57	91.88 ± 1.20	95.00 ± 1.44	92.50 ± 2.70	92.50 ± 1.02	0.733
Average initial weight (g)	14.66 ± 0.37	15.13 ± 1.23	14.60 ± 0.60	15.05 ± 0.25	14.81 ± 0.56	0.975
Average final weight (g)	74.99 ± 1.51 ^b^	75.77 ± 1.88 ^b^	81.42 ± 0.72 ^a^	76.38 ± 1.32 ^b^	77.18 ± 0.82 ^b^	0.029
Total length (cm)	17.59 ± 0.27 ^a^	15.08 ± 0.29 ^c^	16.56 ± 0.38 ^ab^	16.41 ± 0.35 ^b^	16.39 ± 0.52 ^b^	0.001
*K*	1.30 ± 0.01 ^b^	2.04 ± 0.17 ^a^	1.68 ± 0.12 ^ab^	1.58 ± 0.02 ^b^	1.57 ± 0.16 ^b^	0.047
DGC (% BW day^–^^1^)	2.16 ± 0.05	2.10 ± 0.09	2.25 ± 0.04	2.11 ± 0.03	2.15 ± 0.03	0.317
FR (% BW day^−1^)	2.42 ± 0.11	2.48 ± 0.05	2.28 ± 0.06	2.46 ± 0.08	2.43 ± 0.03	0.301
FCR (g feed g gain^−1^)	1.70 ± 0.08	1.64 ± 0.09	1.71 ± 0.09	1.69 ± 0.07	1.83 ± 0.08	0.635
PER (g gain g protein^−1^)	1.93 ± 0.10	2.06 ± 0.11	1.93 ± 0.11	1.97 ± 0.08	1.83 ± 0.08	0.584

FPKM, fermented palm kernel meal; *K*, Fulton’s condition factor; DGC, daily growth coefficient; BW, body weight; FR, feeding rate; FCR, feed conversion ratio; PER, protein efficiency ratio. Data are expressed as mean ± SEM of all fish in three replications. Significant differences in each row are indicated by different superscripts (*p* < 0.05).

**Table 4:** Digestive enzyme activity of sex-reversed red tilapia fed with experimental diets containing varying levels of FPKM for 12 weeks.

Digestive Enzyme	0FPKM	25FPKM	50FPKM	75FPKM	100FPKM	*p*-Value
Pepsin (U mg protein^−1^)	0.50 ± 0.10 ^b^	0.60 ± 0.19 ^b^	1.42 ± 0.17 ^a^	1.24 ± 0.15 ^a^	0.46 ± 0.13 ^b^	<0.001
Trypsin (mU mg protein^−1^)	165.34 ± 8.21 ^c^	158.09 ± 10.48 ^c^	205.98 ± 12.29 ^b^	142.60 ± 18.05 ^c^	258.27 ± 17.77 ^a^	<0.001
Amylase (U mg protein^−1^)	59.25 ± 2.58	52.96 ± 2.11	55.62 ± 2.74	61.93 ± 3.57	52.14 ± 2.03	0.056
Cellulase (U mg protein^−1^)	227.70 ± 11.21 ^c^	241.09 ± 16.41 ^c^	265.18 ± 16.01 ^bc^	268.56 ± 18.24 ^ab^	314.83 ± 10.18 ^a^	0.002
Lipase (mU mg protein^−1^)	42.00 ± 3.41 ^b^	40.72 ± 3.75 ^b^	45.11 ± 2.31 ^b^	61.00 ± 2.64 ^a^	53.95 ± 3.22 ^ab^	0.012

FPKM, fermented palm kernel meal. Data are expressed as mean ± SEM (*n* = 9). Significant differences in each row are indicated by different superscripts (*p* < 0.05).

**Table 5:** Protein synthesis capacity and amount of myosin and actin in white muscle of sex-reversed red tilapia observed at the end of 12 weeks of dietary treatment.

Flesh Parameter	0FPKM	25FPKM	50FPKM	75FPKM	100FPKM	*p*-Value
RNA (μg g^−1^)	3,612 ± 123 ^a^	3,614 ± 93 ^a^	3,604 ± 141 ^a^	3,052 ± 113 ^b^	3,077 ± 171 ^b^	0.002
Protein (mg g^−1^)	199.97 ± 16.45 ^b^	221.65 ± 12.19 ^ab^	240.51 ± 7.26 ^a^	197.58 ± 7.69 ^b^	216.25 ± 12.91 ^ab^	0.046
RNA/protein ratio (μg mg^−1^)	18.74 ± 1.64 ^a^	16.54 ± 0.81 ^ab^	15.12 ± 0.80 ^b^	14.43 ± 1.12 ^b^	14.45 ± 0.93 ^b^	0.045
∆H_Myosin_ (J g^−1^)	0.67 ± 0.08	0.57 ± 0.03	0.80 ± 0.19	0.80 ± 0.07	0.68 ± 0.07	0.485
∆H_Actin_ (J g^−1^)	0.34 ± 0.00	0.23 ± 0.03	0.34 ± 0.07	0.25 ± 0.05	0.31 ± 0.01	0289
∆H_Myosin + Actin_(J g^−1^)	1.07 ± 0.07	0.81 ± 0.06	0.97 ± 0.20	1.06 ± 0.10	1.07 ± 0.08	0.330
∆Actin/myosin ratio	0.58 ± 0.06 ^a^	0.41 ± 0.03 ^ab^	0.54 ± 0.01 ^a^	0.32 ± 0.06 ^b^	0.47 ± 0.06 ^ab^	0.050

FPKM, fermented palm kernel meal; ∆H, protein denaturation enthalpy. Data are expressed as mean ± SEM (*n* = 9). Significant differences in each row are indicated by different superscripts (*p* < 0.05).

**Table 6:** Whole body composition (% of wet weight basis) of sex-reversed red tilapia fed with experimental diets containing varying levels of FPKM for 12 weeks.

Composition (%)	0FPKM	25FPKM	50FPKM	75FPKM	100FPKM	*p*-Value
Moisture	67.48 ± 0.77	66.71 ± 0.68	68.15 ± 0.85	67.29 ± 0.72	67.96 ± 0.71	0.719
Crude protein	17.26 ± 0.10	13.09 ± 4.58	15.30 ± 1.72	17.64 ± 0.44	18.49 ± 0.32	0.465
Crude lipid	5.94 ± 0.72	6.42 ± 0.54	4.77 ± 0.60	4.88 ± 0.59	5.17 ± 0.62	0.309
Ash	4.99 ± 0.35	5.45 ± 0.52	5.26 ± 0.60	5.59 ± 0.40	5.72 ± 0.47	0.795

FPKM, fermented palm kernel meal. Data are expressed as mean ± SEM (*n* = 9). Significant differences in each row are indicated by different superscripts (*p* < 0.05).

**Table 7:** Hematological parameters of sex-reversed red tilapia fed with experimental diets containing varying levels of FPKM for 12 weeks.

Hematological Parameter	0FPKM	25FPKM	50FPKM	75FPKM	100FPKM	*p*-Value
RBC (×10^6^ cells μL^−1^)	1.95 ± 0.07	1.95 ± 0.13	2.05 ± 0.07	2.04 ± 0.05	2.00 ± 0.13	0.901
WBC (×10^4^ cells μL^−1^)	37.33 ± 2.52 ^b^	38.37 ± 4.48 ^b^	55.67 ± 2.73 ^a^	52.90 ± 1.87 ^a^	59.20 ± 4.42 ^a^	0.002
Hb (g dL^−1^)	7.33 ± 0.18	7.60 ± 0.44	7.78 ± 0.23	7.78 ± 0.23	7.33 ± 0.25	0.637
Hematocrit (%)	28.88 ± 0.64	29.93 ± 1.75	30.83 ± 0.93	31.05 ± 0.96	28.88 ± 1.09	0.526
MCH (pg cell^−1^)	37.70 ± 0.40	39.08 ± 0.61	37.98 ± 0.86	38.05 ± 0.50	38.50 ± 0.31	0.517
MCHC (g dL^−1^)	25.35 ± 0.06 ^a^	25.40 ± 0.06 ^a^	25.20 ± 0.07 ^ab^	25.05 ± 0.05 ^b^	25.38 ± 0.11 ^a^	0.021
MCV (fL)	148.50 ± 1.85	154.00 ± 2.71	150.75 ± 3.25	152.00 ± 2.12	151.67 ± 1.86	0.618
Lymphocyte (%)	90.33 ± 1.45	77.33 ± 9.82	73.25 ± 8.16	81.75 ± 3.09	82.00 ± 4.55	0.437
Monocyte (%)	4.50 ± 1.19	6.50 ± 1.19	6.00 ± 1.47	4.00 ± 0.58	5.75 ± 1.75	0.694
Neutrophil (%)	17.50 ± 11.86	25.00 ± 12.21	20.75 ± 6.77	12.00 ± 3.94	12.25 ± 5.36	0.799
Plasma protein (g dL^−1^)	1.44 ± 0.13 ^c^	2.13 ± 0.13 ^b^	2.03 ± 0.05 ^b^	1.91 ± 0.11 ^b^	2.54 ± 0.12 ^a^	<0.001
BUN (mg dL^−1^)	6.50 ± 1.55	6.00 ± 1.35	5.75 ± 1.25	2.67 ± 0.33	4.75 ± 1.31	0.365
Creatinine (mg dL^−1^)	0.46 ± 0.37	0.24 ± 0.08	0.64 ± 0.28	0.59 ± 0.21	0.30 ± 0.12	0.696
Uric acid (mg dL^−1^)	0.85 ± 0.59	1.25 ± 0.62	1.48 ± 1.09	0.23 ± 0.05	0.63 ± 0.28	0.655
ALP (U L^−1^)	39.33 ± 7.97	33.00 ± 4.06	29.25 ± 5.04	29.33 ± 2.85	25.67 ± 5.78	0.509
AST (U L^−1^)	124.33 ± 12.57	92.67 ± 9.91	128.50 ± 17.50	114.67 ± 5.50	70.00 ± 14.74	0.181

FPKM, fermented palm kernel meal; RBC, red blood cells; WBC, white blood cells; Hb, hemoglobin; MCH, mean corpuscular hemoglobin; MCHC, mean corpuscular hemoglobin concentration; MCV, mean corpuscular volume; BUN, blood urea nitrogen; ALP, alkaline phosphatase; AST, aspartate aminotransferase. Data are expressed as mean ± SEM (*n* = 9). Significant differences in each row are indicated by different superscripts (*p* < 0.05).

## Data Availability

Data are available on request from the authors.
